# Three-dimensional printed models can reduce costs and surgical time for complex proximal humeral fractures: preoperative planning, patient satisfaction, and improved resident skills

**DOI:** 10.1186/s10195-024-00754-6

**Published:** 2024-02-28

**Authors:** Andrea Fidanza, Gianfilippo Caggiari, Francesco Di Petrillo, Enrico Fiori, Alberto Momoli, Giandomenico Logroscino

**Affiliations:** 1 Unit of Orthopaedics, Department of Life, Health and Environmental Sciences, University of L’Aquila (IT), Piazzale S.Tommasi, 1, 67100 L’Aquila, Italy; 2 Orthopaedic and Traumatology Department, Sassari University Hospital, Sassari, Italy; 3 Unit of Trauma and Orthopaedic, San Bortolo Hospital, Vicenza, Italy

**Keywords:** Three-dimensional printing, 3D models, Rapid prototyping, Computer-aided trauma surgery, Humerus fracture, Education, Training, Surgical cost, Planning, Informed consent

## Abstract

**Background:**

Proximal humeral fractures (PHFs) are still controversial with regards to treatment and are difficult to classify. The study’s objective is to show that preoperative planning performed while handling a three-dimensional (3D) printed anatomical model of the fracture can ensure a better understanding of trauma for both surgeons and patients.

**Materials and methods:**

Twenty patients (group A, cases) with complex PHF were evaluated preoperatively by reproducing life-size, full-touch 3D anatomical models. Intraoperative blood loss, radiographic controls, duration of surgery, and clinical outcomes of patients in group A were compared with 20 patients (group B, controls) who underwent standard preoperative evaluation. Additionally, senior surgeons and residents, as well as group A patients, answered a questionnaire to evaluate innovative preoperative planning and patient compliance. Cost analysis was evaluated.

**Results:**

Intraoperative radiography controls and length of operation were significantly shorter in group A. There were no differences in clinical outcomes or blood loss. Patients claim a better understanding of the trauma suffered and the proposed treatment. Surgeons assert that the planning of the definitive operation with 3D models has had a good impact. The development of this tool has been well received by the residents. The surgery was reduced in length by 15%, resulting in savings of about EUR 400 for each intervention.

**Conclusions:**

Fewer intraoperative radiography checks, shorter surgeries, and better patient compliance reduce radiation exposure for patients and healthcare staff, enhance surgical outcomes while reducing expenses, and lower the risk of medicolegal claims.

**Level of evidence:**

Level I, prospective randomized case–control study.

## Background

Anatomical three-dimensional (3D) printers are a type of technology that can duplicate solid objects using digital scans from informatics. These samples are utilized in medical research to examine anatomical deformities that have a significant impact on surgical practice [1–4].

In the trauma field, a 3D-printed anatomical model may offer a direct and interactive image of the characteristics of a fracture, assisting orthopedic surgeons in better operational planning [1]. Additionally, this technology can be used to simulate surgical procedures, such as fracture reduction and hardware selection to get the best fixation, just as has been done for some anatomical areas, including acetabulum, wrist, tibia, and calcaneus [5–7].

Complex proximal humeral fractures (PHFs) are still subject to significant controversy regarding treatment, and it is difficult to effectively classify this type of injury [8–10]. The general consensus is that, since fracture classification alone is not sufficient to determine treatment, each case should be assessed individually, studying calcar involvement to propose the surgical management (reconstruction versus prosthesis) [11]. According to a Cochrane Review [12], open reduction and internal fixation (ORIF) is the gold standard in Neer III or IV proximal humerus fractures [13] if vascular integrity is preserved [14, 15].

The authors’ hypothesis is that handling a 3D-printed model of a PHF could ensure more rigorous preoperative planning, reduce surgical time, and gain a better understanding of trauma for both clinicians and patients.

## Materials and methods

A prospective, randomized, double-center, case–control study was carried out from July 2019 to July 2021. Applying the same protocol, two neighboring hospitals were involved in this study: a university hospital and a public hospital. Patients were enrolled by meeting these inclusion criteria: age from 18 to 90 years old; recent thee- or four-part proximal humeral fracture; surgical reconstruction with ORIF (plate and screws). Exclusion criteria were: polytrauma patients with life-threatening injuries, dementia, life expectation less than 6 months, pathological fractures, surgical indication to nail, or prosthesis.

All patients were completely informed about surgical procedure, treatments, possible complications, and therapeutic alternatives. The study was approved by the internal review board.

All patients underwent routine X-ray and two dimensional (2D) computed tomography (CT) scans (Aquilion One, Toshiba Medical System) and 3D volume renderings to investigate possible complications or alternative diagnostic outcomes. The volumetric acquisition was performed with a slice thickness of 0.5 mm.

**Fig. 1:**
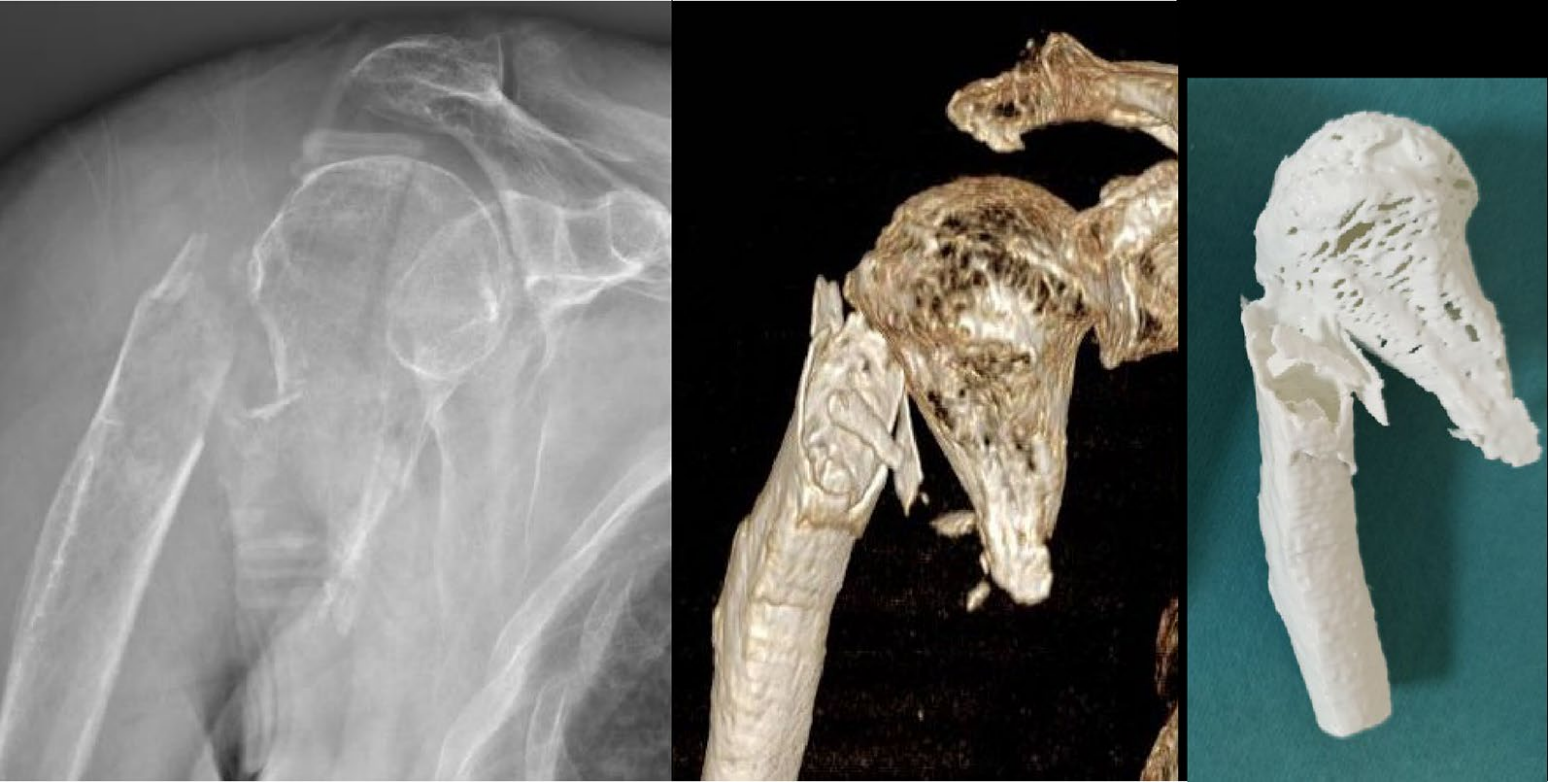
From radiography to CT with 3D volumetric rendering to full-size 3D-printed fracture replica: the transformation of surgical planning from the virtual stage to the real stage

Patients who met our inclusion criteria were enrolled from both hospitals and randomly divided into two groups using the free random.org online app. Group A patients (cases) were assessed with a more in-depth investigation of the fracture from the time of admission to the emergency room: full-touch, real-size 3D anatomical models were generated using CT images (Fig. 1). On the contrary, only X-ray and CT scan were used for the standard preoperative evaluation of patients in group B (controls), following Association of Osteosynthesis (AO) trauma planning principles, studying CT scan, and drawing the preoperative plan on an anteroposterior view on paper.

Utilizing a well-defined methodology to create a 1:1 scale 3D-printed model using polytactic acid (PLA) plastic filament [1], two full-touch anatomical models were created for each patient in group A: a “fracture model” and a “reduction model.” Our academic institution owns the printer, which is made freely available for research purposes. The “fracture model” served as a static representation of the fracture, in which artificial “3D bridges” were included to stabilize and maintain the precise and current positions of the bone fragments. To replicate the spongy tissue, polyurethane foam was injected into the PLA bones of the “reduction model” during the post-production process.

### In vitro surgery

**Fig. 2:**
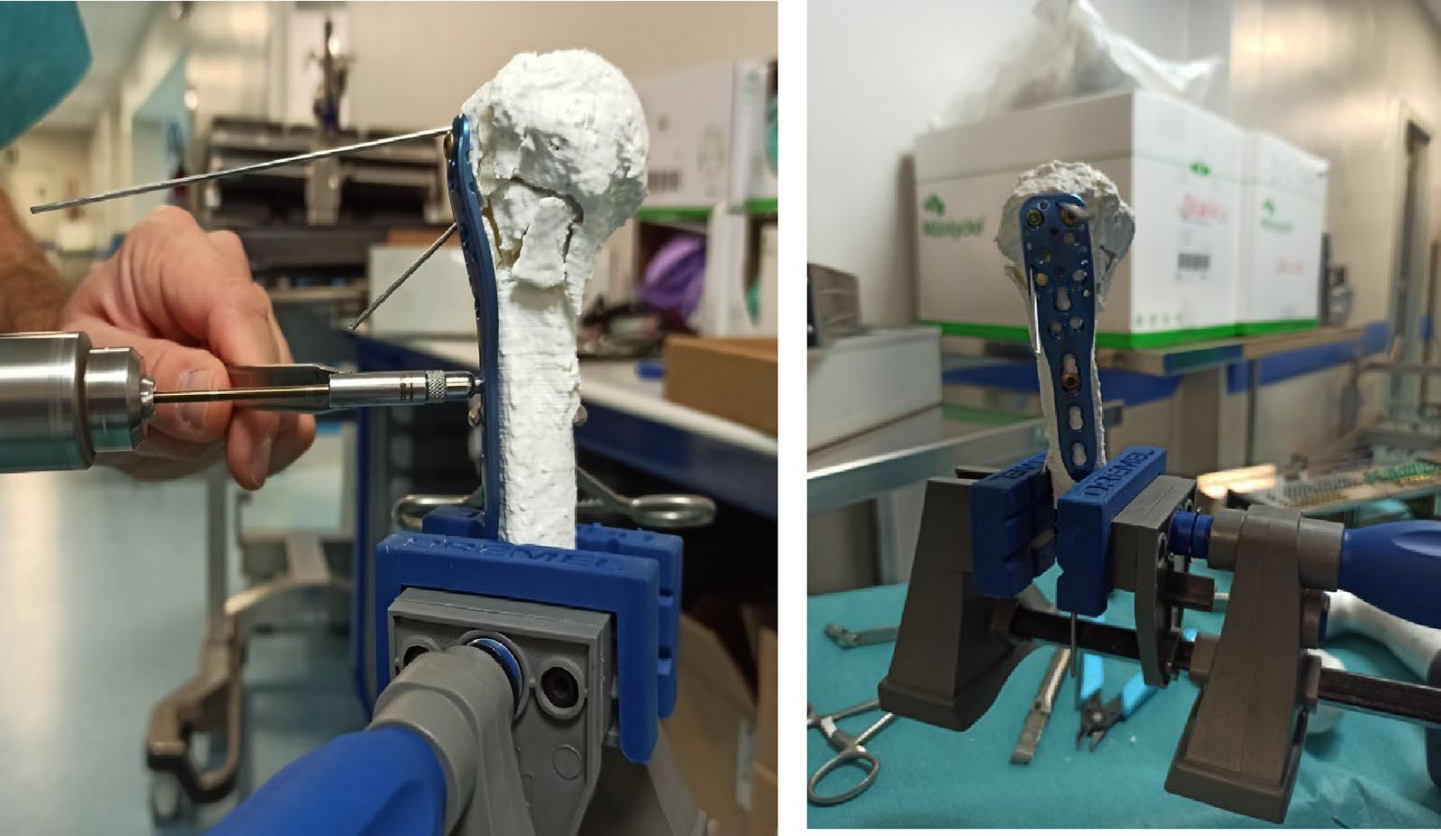
Residents and senior surgeon perform “in vitro” planning and on-the-table simulation of the surgery

Once the 3D replica of the fracture was completed, the senior surgeon and residents were able to touch the full-size construct the day before surgery, which allowed them to fully understand the pattern of fracture, the displacement of the fragments, the impact of the head. The correct procedure to reduce the fragments using the “reduction model” was then simulated on-the-table, and the proper size and location of the plate, length, and orientation of the screws were chosen and used (Fig. 2).

**Fig. 3:**
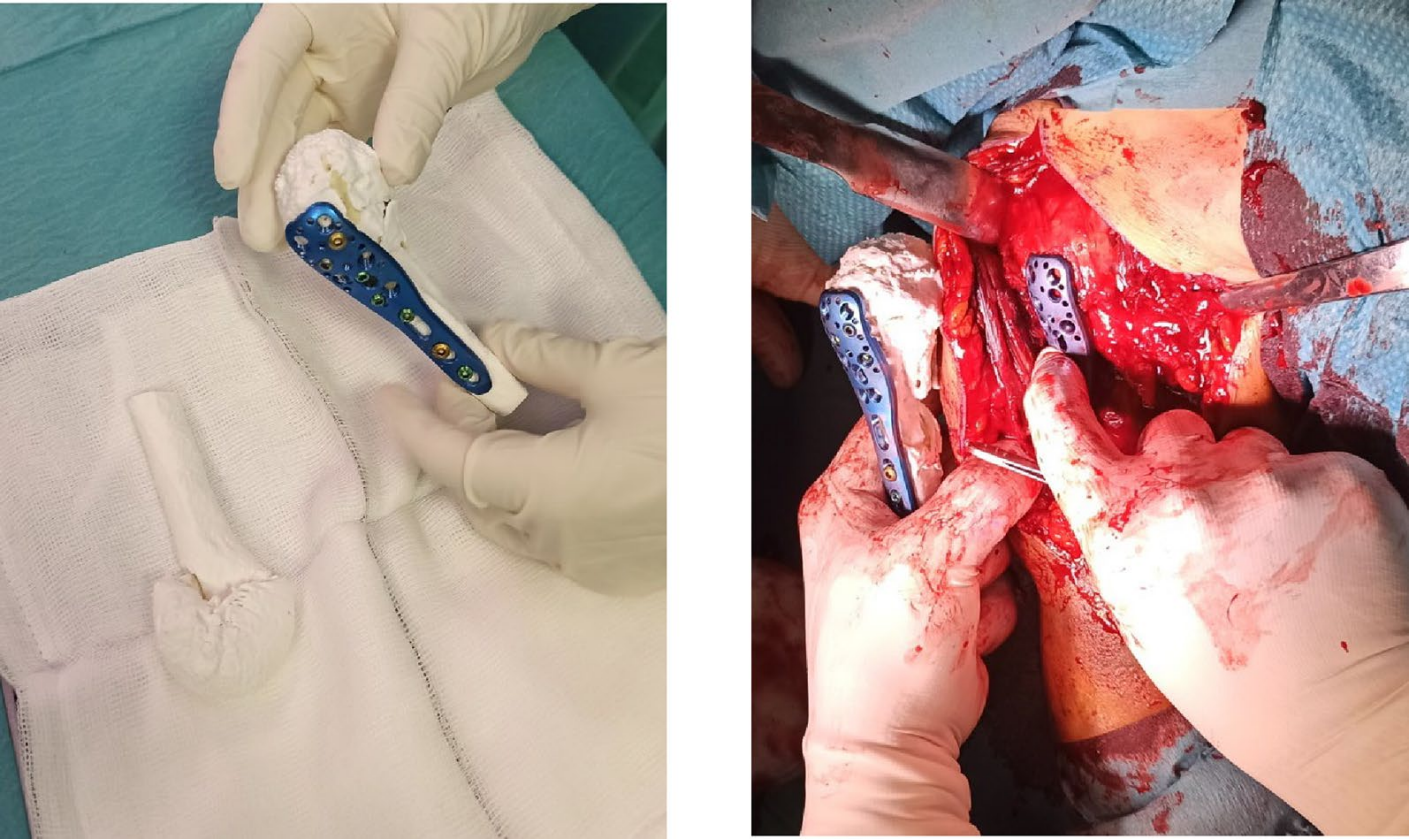
Both the “fractured” and “reduced” 3D printed full-touch models were steam sterilized for handling during surgery

Both the “fracture” and “reduced” 3D models were steam sterilized for 45 min at 121 °C before being introduced into the operating room. During the operation, the surgeon can handle the models to verify the position of the hardware, expedite the movement sets, and minimize potential complications (Fig. 3).

### Measurements

It was assessed how group A and group B differed in terms of the length of the procedure (from the incision to the skin suture), the quantity of intraoperative X-ray checks, and intraoperative blood loss (calculated by subtracting the lavage fluids from the total amount of the suction container and the weight of the soaked gauze after subtracting the weight of the dry gauze) [16]. In both groups, the planned fracture reduction, the size and positioning of the plate, the holes used, and the direction of the screws were compared with the final fixation; in group A the length of the screws used in 3D planning was also compared with those implanted. The Constant–Murley Shoulder Score (CSS) [17] was used to evaluate the clinical outcomes at 1, 3, 6, and 12 months and then annually.

### Patient compliance and surgeon satisfaction

Patients in group A participated in improving doctor–patient communication by showing them both the “fracture model” and the “reduction model” already fixed with plate and screws. This was done to make the patient understand the severity of the damage suffered and the postoperative expectations and thereby increase compliance. Informed consent to the surgery was also amended for this set of patients, adding that the patient had fully comprehended the damage sustained and the proposed procedure by handling the 3D plastic replicas themselves.

Patients in group A were interrogated via questionnaire, already validated by Samalia et al. [18] to obtain feedback on the use of anatomical modelling. Similarly, the surgeons who performed the operations (two residents and a senior surgeon per hospital) also responded to a questionnaire to determine the benefits and utility of anatomical models in comparison with preoperative information gathered via X-rays and CT scans. The understanding level was rated on a score between 1 and 10. Patients and senior surgeons answered five questions. To focus the questionnaire on education, two more specific questions regarding training were included in the resident survey. Residents were asked to complete the questionnaire assuming they were the surgeon who was the main operator to perform the operation. Before surgery, the doctors provided answers to questions 1 and 2. The other answers were provided at the end of the intervention.

### Cost analysis

The cost analysis was done for both direct and indirect surgical activity costs [18, 19]. While the surgery time was regarded as an indirect cost, the manufacture of the 3D-printed models and sterilizing were considered direct expenditures. The cost of an active operating room for major shoulder surgery was calculated on the basis of a report by the Italian Society of Orthopedics and Traumatology (SIOT) [20].

### Statistical analysis

The statistical analysis was based on an estimated sample size of at least 30 subjects, with a ratio 1:1 for the two treatment groups, which was calculated to be adequate to achieve 90% power to detect a large effect size (Cohen’s *f* of 0.40). Data were collected in a database and analyzed using the SAS System version 9.4 (SAS, Cary, NC USA).

Student’s *t* test, chi-squared test, and Fisher’s exact test were performed as appropriate to evaluate the initial differences between the randomized groups. The differences between CSS, the number of intraoperative radiographs, blood loss, and surgical times between group A and group B were analyzed with Student’s *t* test. Responses to questionnaires for quantitative data were assessed with Wilcoxon’s signed rank test. The values are reported as the means of the results detected by the measurements studied ± standard deviation (SD). Values were considered significant if *p* ≤ 0.05.

## Results

The study included 40 patients equally divided into group A and group B (10 patients from the university hospital plus 10 from the public hospital per group). The minimum follow-up was 2 years (24–36 months). The fractures healed without complications in the total sample. The surgical team in each hospital was always the same: a senior surgeon and two residents. The four residents involved are all enrolled in the same postgraduate institution. General narcosis or brachial plexus anesthesia were used. The PHILOS plate (Synthes, Paoli, PA, USA) was used in all operations, which were carried out while the patient was sitting in a beach-chair position, performing a pectoral-deltoid approach [9].

The demographic composition is described in Table 1, resulting in an initial lack of differences between the two groups for sex (*p* = 0.287), age (*p* = 0.425), and fracture classification (*p* = 0.312), and demonstrating homogeneous distribution of the sample.

**Table 1:** The sample: the 40 patients were randomly divided into two groups

	Group A			Group B		
	Sex*	Age**	Neer***	Sex*	Age**	Neer***
*Public hospital*						
Patient 1	F	62	III	M	72	IV
Patient 2	F	73	IV	F	63	III
Patient 3	F	76	III	M	78	III
Patient 4	M	76	IV	M	63	III
Patient 5	F	65	III	F	71	IV
Patient 6	M	73	III	F	78	III
Patient 7	F	71	IV	M	76	IV
Patient 8	F	66	III	F	75	III
Patient 9	M	72	III	F	74	IV
Patient 10	F	71	IV	M	67	III
*University hospital*						
Patient 1	M	69	IV	F	69	IV
Patient 2	F	76	III	F	74	III
Patient 3	M	76	III	M	65	IV
Patient 4	F	65	III	F	68	III
Patient 5	F	72	IV	M	71	III
Patient 6	M	76	III	F	74	III
Patient 7	F	72	IV	F	79	IV
Patient 8	F	67	III	F	65	III
Patient 9	M	73	III	F	72	IV
Patient 10	F	68	III	M	69	III
Total	13 F 7 M	70.4 ± 4.2	13 III and 7 IV	12 F and 8 M	71.1 ± 5.3	12 III and 8 IV

*Fisher’s exact test (*p* = 0.287)

**Student’s *t* test (*p* = 0.425)

***Chi-squared test (*p* = 0.312)

The average of the measurements reported is the result of the sum of the patients enrolled in both hospitals. The results divided by single hospitals are available upon specific request to the corresponding author. The mean length of surgery was 75.47 ± 9.06 min in group A and 88.55 ± 11.20 min in group B. The procedures were on average 15% shorter using 3D anatomical modeling (*p* = 0.0002). A higher number of intraoperative X-ray checks (*p* = 0.0001) were performed in patients in group B (12.24 ± 2.1) when compared with group A (9.44 ± 2.0). Blood loss did not differ between groups A and B (*p* = 0.8633): the average loss was 268.75 ± 67.81 mL in group A and 272.02 ± 50.18 in group B. At the last follow-up, no differences were found in clinical results between the two groups: the mean CSS score for patients in group A was 70 (47–87) ± 11.9, and it was 72 (66–83) ± 5.7 in group B (*p* = 0.5020; Table 2). In both groups, the postoperative reduction of the fracture, the size of the plate, and the choice of holes to be used respected the preoperative planning. In addition, in group A, the plate seating and the number and the length of the screws previously applied on the 3D model were also compared, resulting in a substantial overlap. In particular, the length of the screws used during the simulation with those finally implanted showed a difference in the final implantation of ±2 mm exclusively in the cephalic screws, while the diaphyseal screws were identical.

**Table 2:** Measurements of patients in group A (planning with 3D anatomical models created from CT images) compared with group B (standard preoperative investigations: X-ray + CT)

Measurements	Group A	Group B	*p*-Value
Length of surgery (min)	75.47 ± 9.06	88.55 ± 11.20	0.0002
Intraoperative X-ray checks	9.44 ± 2.00	12.24 ± 2.10	0.0001
Blood loss (mL)	268.75 ± 67.81	272.02 ± 50.18	0.8633
Constant shoulder score	70.00 ± 11.90	72.00 ± 5.70	0.502

The definitive answers to the questionnaire are reported in Table 3 (patients survey), in Table 4 (senior surgeons survey), and in Table 5 (residents survey). After the 3D models had been shown, the mean patient understanding score increased by an average of 2.4 points, from 5.6 to 8.00 (*p* < 0.05). Each patient had never seen a 3D representation of a specific body part before. The majority of patients (85%) supported routinely using 3D printing to explain the suggested intervention (Yes 17, No 3). The surgeon-related score increased by 1.4 (*p* < 0.05) from 8.1 without the usage of the models to 9.5 after their adoption. Surgeons preferred further use of this technology, and in almost all cases, they noted that the 3D-printed model added valuable details for planning and surgical procedure. Finally, no resident had ever used a 3D-printed fracture model to simulate a reduction and osteosynthesis operation. Additionally, they claimed that the surgery helped them feel more confident if they could always simulate it initially (scoring 8.3 out of 10).

**Table 3:** Summary of patient survey results (20 patients interviewed)

Patient questionnaire	Public hospital			University hospital		
	Average	Median	DS	Average	Median	DS
1. Have you ever seen a 3D-printed model of any part of your body before? (yes/no)	Yes = 0, 0% No = 10, 100%			Yes = 0, 0% No = 10, 100%		
2. How well do you understand the severity of your fracture via images and RX? (1–10)	5.4	5	1.02	5.8	6	1.17
3. How well do you understand the severity of your fracture with a 3D model? (1–10)	8.2	8	0.75	7.8	8	0.75
4. How critical has it been to see a physical model of your fracture? (1–10)	6.2	6	1.60	6.2	6	2.04
5. Do you suggest producing and obtaining a 3D-printed model before operation? (yes/no)	Yes = 9, 90% No = 1, 10%			Yes = 8, 80% No = 2, 20%

**Table 4:** Summary of senior surgeons survey results (two surgeons interviewed)

Senior surgeon questionnaire	Public hospital			University hospital		
	Average	Median	SD	Average	Median	SD
1. How reliable was the diagnosis of the articular damage (comminution and number of fragments) based only on computerized images? (1–10)	8	8	1.26	8.2	8	0.98
2. How reliable was the diagnosis of the articular damage (comminution and number of fragments) after handling the 3D-printed model? (1–10)	9.6	10	0.49	9.4	10	0.80
3. Did the availability of the 3D-printed model influence your surgical indication? (yes/no)	Yes = 2, 20% No = 8, 80%			Yes = 10, 100% No = 0, 0%		
4. Did the 3D-printed model influence implant selection? (yes/no)	Yes = 10, 100% No = 0, 0%			Yes = 10, 100% No = 0, 0%		
5. Would you use 3D-printed models for other fractures and would you suggest their use to any of your colleagues? (yes/no)	Yes = 10, 100% No = 0, 0%			Yes = 10, 100% No = 0, 0%

**Table 5:** Summary of resident survey results (four residents interviewed)

Resident questionnaire	Public hospital			University hospital		
	Media	Average	DS	Media	Average	DS
1. How reliable was the diagnosis of the articular damage (comminution and number of fragments) based only on computerized images? (1–10)	7.3	7	1.61	7.4	7.5	1.62
2. How reliable was the diagnosis of the articular damage (comminution and number of fragments) after handling the 3D-printed model? (1–10)	9	9	0.77	9.1	9	0.70
3. Did the availability of the 3D-printed model influence your surgical indication? (yes/no)	Yes = 6, 30% No = 14, 70%			Yes = 2, 10% No = 18, 90%		
4. Did the 3D-printed model influence implant selection? (yes/no)	Yes = 20, 100% No = 0, 0%			Yes = 20, 100% No = 0, 0%		
5. Would you use 3D-printed models for other fractures and would you suggest their use to any of your colleagues? (yes/no)	Yes = 20, 100% No = 0, 0%			Yes = 20, 100% No = 0, 0%		
6. Have you ever simulated a trauma surgery with a 3D-printed model? (yes/no)	Yes = 0, 0% No = 20, 100%			Yes = 0, 0% No = 20, 100%		
7. How much security and confidence would you have with surgery if you could always simulate it first? (1–10)	8.3	8.5	0.78	8.3	8	0.64

Preoperative planning in group A was more intricate than in group B. The time required to complete the replica of the natural-size model may range from as little as 4 h in simple samples to as long as 11 h for complex ones.

The direct costs are represented by the material used to create the models (usually the extruded PLA filament costs less than EUR 5 per piece). There was no additional cost associated with sterilizing the 3D models, nor was there a difference between group A and group B in the number of sterilized devices used during procedures. The hardware used (laptop, driver, and printer) is basic, and in our experience had been purchased in advance by the university (at a cost of approximately EUR 1000), therefore they are not calculated in the costs of this study. The software used is available free of charge for noncommercial purposes (InVesalius software version 3.1; Center for Information Technology Renato Archer, MCTI, Brazil).

In 2009, the Italian Society of Orthopedics and Traumatology presented a budget analysis with diagnostic related group (DRG) costs and ministerial costs. The data showed an average cost of EUR 1963 per h for an active operating room in major shoulder/elbow surgery (DRG 223). With a mathematical formula, we calculated the cost of the active operating room per min, thus obtaining the final expense for the average duration of the surgery: (1963: 60) × 75.47 = 2469 EUR in group A; (1963: 60) × 88.55 = 2897 EUR in group B. The reduction in operative length and the use of the theater by about 15% have generated savings of up to EUR 428 (2897–2469) for each single operation in patients in group A.

## Discussion

Personalized medicine in surgery represents a continuous search for improvement, reducing risks and human errors [21, 22]. As a result of the revolutionary changes in orthopedic and trauma diagnoses brought about by the discovery of X-rays in 1895, as of now, digital technologies and 3D printing are so widespread and well-liked that they are widely used in both trauma and arthroplasty [23, 24]. The main findings of this paper are that a 3D replica of a PHF can assist surgeons in diagnosing, planning, and performing ORIF, increase patient compliance, and improve residents’ satisfaction. Furthermore, surgical simulation can lead to a clear reduction in the duration of the operation.

Scientific attention toward the use of 3D-printed replicas has increased a lot in recent years, however the application in the daily routine of this technology for traumatic joint bone injuries is less widespread and common [7, 23]. This discrepancy may be due to difficulties in organizing the workflow of 3D printing organizations (emergency, radiologic, and orthopedic departments), as well as the availability of equipment and resources [18]. In our experience, having software and hardware already in place has allowed us to convert to an .stl file instantly and automatically after the CT scan: this approach allows the model to be available within a maximum of 11 h, providing time to use it to plan and perform an early surgery when necessary. Moreover, another strong point of this study is that we intended to concretely evaluate the feedback of this procedure both from the point of view of the patient and the surgeons. The results derived from our experience are encouraging: the randomization of the sample allowed us to compare the surgical times, the blood loss, and the quantity of intraoperative X-rays performed. In patients in group A, the lower number of X-ray controls performed and the significant reduction in the duration of the operation, even if not related to a significantly lower blood loss, suggest the advantage that can be derived from a more detailed observation of the fracture. This method allows us to fully know the comminution and dislocation of the fragments, to anticipate the potential bone defects, and therefore to predict the need for a bone graft, transforming a theoretical simulation of surgical planning from the virtual stage to the real stage [25].

At the same time, we did not expect a significant improvement in clinical outcomes in patients who underwent 3D preoperative planning. The substantial overlap of functional outcomes at a 2-year follow-up is likely due to the fact that surgery in each case was performed by an experienced surgeon. A recent review and meta-analysis of PHF treated with plate and screws shows that the final CSS of these patients is 75 ± 15.8 points [9]. In our case, the results of both groups intersected the standard deviation of the aforementioned meta-analysis (group A: CSS = 70 ± 11.9; group B: CSS = 72 ± 5.7), without observing any particular differences.

It is always difficult for patients and their families to fully understand the severity of the fracture and expected outcomes. None of the 20 patients had ever seen a 3D-printed model of any part of their body previously, as evidenced by the answers to our questionnaire. Although we have not been involved in any legal disputes, which is also a result of the absence of complications in this set of patients, we are confident that doctor–patient and family practice communication can become a useful tool for boosting understanding and compliance both before surgery and during recovery. We think the ability to amend the informed consent wording after giving the patient access to a natural-size replica of their fracture and ensuring that the proposed surgery is clearly understood should also lessen the likelihood of a complaint stemming from clinical dissatisfaction.

In general, the interpretation of imaging data is now actually a process of integrating 2D and 3D images, but in complex fractures of the proximal humerus, with difficult-to-understand spatial structures, the analysis and complete determination of the fracture pathological anatomy are more difficult [8, 11, 26]. The occurrence of iatrogenic complications is not only associated with surgical skill and mastery of theoretical knowledge, but also related to preoperative diagnosis and surgical planning [1]. This is especially true for junior surgeons and trainees, where hands-on practical experience goes a long way in successfully performing high-demand surgeries [27, 28]. In addition, a full-touch fracture model appears to be essential to facilitate communication among surgical team members, thus improving performance and collaboration. In particular, the senior surgeons who answered question no. 4 commented that the choice of plate’s holes and the direction of the screws was influenced by the in vitro surgery. Moreover, they greatly appreciated the possibility of handling the sterilized model during surgery, substantially since it will allow them to avoid any inconveniences or diversions that might arise during the interpretation of X-ray and CT images. In answer to question no. 5, they suggested implementing the use of 3D models for other complex and comminuted fractures, advocating not only greater understanding of fractures but also ease of surgical planning.

Also from a didactic point of view this method may represent a useful option to study anatomy regarding the review of surgical techniques and fractures [28]. In particular, the residents were allowed to practice, improving their skills and learning curve, and they aimed for more widespread utilization of modern and innovative tools such as this one, to approach major surgery with more confidence. In place of the more expensive cadaver lab activities, the simulation of the surgical act using 3D printing, virtual, augmented, and mixed reality may be a good alternative [29]. These findings, along with the opinions of the residents, have led us to incorporate this technology into our university’s residency program, as has already been done in other structures [18, 30]. Basically, owning an institutional 3D printer can prove to be an important training tool during residency programs [27, 28, 30].

However, the cost-effectiveness analysis is what determines whether this technology can be developed and is even feasible. A 3D-printed model adds to the already mandatory costs of X-ray or CT examinations. One of the most innovative approaches to measure expenses more accurately and address cost challenges is time-driven activity-based costing. However, this sophisticated instrument is equally suitable in the orthopedic field, at least in the trauma field, owing to the poor reproducibility of surgery [19, 31]. The calculation of the capacity cost rate (CCR), which is the practical capacity of each active operator providing procedures, is not suitable for short interventions and with healthcare workers on fixed salaries [15]. For this reason, we used the more traditional tool of comparing direct and indirect costs, excluding from the result the mandatory expenses incurred by all patients (such as hospitalization, tests, and overhead expenses). In our experience, the sterilization of the devices has not resulted in any additional direct expense, as well as the software used which is free online for noncommercial purposes. Likewise, the hardware (laptop, drivers, and printer) were already owned by our Institute. The direct cost for the reproduction of the “fracture model” and the “reduction model” was EUR 5 each (EUR 10 per patient). The size of the pieces, the type of resin used for printing, and the details of the prototypes can directly affect the final cost. The choice of PLA as the first material for 3D model reconstruction mainly depends on its characteristics. It is inexpensive and represents a valid alternative to petroleum-based materials. It has a low fusion point, and compared with other materials it is more moldable and its processing is less toxic and harmful than other cheap materials (Spectrum Group Ltd.—Pęcice, Polska) [1]. Regarding indirect expenditures (active operating room), we observed a definite reduction in operation length (15%) in patients in group A, resulting in a savings of more than EUR 400 per procedure. Despite these encouraging results, it can take some time for health administrations to approve it in daily routine because it is new and not yet acknowledged as a diagnostic tool for understanding fractures in general. In the future, efficiency in terms of time and costs can be anticipated when taking into account a scenario of model production inside a preoperative procedure inserted in routine practice [18].

One of the limitations of this method, however, is the procedures for printing, segmenting, and immobilizing the 3D model, which can be long, tedious, and complex [32]. The quality of CT images, which may produce cuts as small as 0.9 mm [18], is directly related to the precision of 3D printers. Extruded filament used in modern 3D printers has a thickness of roughly 0.1 mm. In truth, the spatial resolution that can be achieved seldom exceeds this limit and is often around 0.5 mm because of the vibrations that are caused by cartridges. Another limitation of surgical simulation compared with real surgery is represented by the absence of the muscular forces, soft tissue, nerves, and veins, which in the 3D model facilitates the implantation of the plate and screw. Furthermore, the sample examined is certainly too small to reach definitive conclusions on the effectiveness of this tool. Finally, even the cost analysis may not be entirely accurate. The document taken as a reference to calculate the savings on indirect costs is from 2009: given inflation and the general rise in healthcare expenditures, the savings might be even higher.

Finally, even the cost analysis may not be entirely accurate. The most recent document for calculating indirect cost savings is from 2009 and does not consider inflation. Even if the savings highlighted by our experience may seem minimal, and the few minutes saved in the duration of the operation can scarcely guarantee the immediate use of the theater for a new operation, the “lack of production” of the healthcare personnel must still be considered: in fact, since in many countries hospitals are considered “commercial companies,” having staff on duty who are not producing leads to economic losses for the administration. However, one of the main limitations of the capacity cost rate is that it must be applied singly in each institution as salaries are potentially different, which is why we did not explore expenses with this tool. Ultimately, another limitation is that we did not consider the time dedicated to planning in the expense account for both groups. This is because surgical simulation, especially on 3D models, must be strongly considered as education and training, and the human resources involved have subjective qualities of reproducibility, skill, and understanding.

## Conclusions

The findings of this study offer both senior and junior surgeons a modern suggestion on the logical planning of an ORIF for complex proximal humerus fractures.

Fewer intraoperative X-ray checks, shorter surgeries, and improved patient compliance all reduce radiation exposure for patients and medical staff, improve surgical results, and lower the possibility of medicolegal lawsuit.

A “new” preoperative planning technique that teaches junior surgeons different surgical approaches may be introduced in the residency program using 3D methodical modeling, which might also improve formative satisfaction and make up for the lack of costly cadaver lab sessions.

## Data Availability

The datasets used and/or analyzed during the current study are available from the corresponding author upon reasonable request.

## Abbreviations

3D: Three-dimensional
PHF: Proximal humeral fractures
ORIF: Open reduction and internal fixation
CSS: Constant shoulder score
SIOT: Italian Society of Orthopaedics and Traumatology
